# 

**Published:** 1883-11

**Authors:** 


					﻿Vol. III.
mww 1883.
No. If.
TPHE
HOMEOPATHIC pH^lClAfl,
A MONTHLY JOURNAL OF MEDICAL SCIENCE.
“ Magna est verilas, el. prcevdlebit.”
' —tL-1- CT_ I_i JbdltLL ZMZ_ _D.,
EDITOR.
CONTENTS:
(See inside of Cover.)
PHILADELPHIA :
No. 2109 CHESTNUT STREET.
3ST BI "W YORK:
A; L. CHATTERTON, PUBLISHING COMPANY, Publisher’s Agents.
X. O 2ST X> O TT :
ALFRED HEATH & CO;, Sole Agents fob Great Britain,
114 Ebury Street, Eaton Square.
Yearly Subscription, $2,50, invariably in advance."
Entered.st the Philadelphia Post-Office as Second-Class Mail Matter.
				

